# Supplementary figures and images for: Photoacoustic imaging to localize indeterminate pulmonary nodules: A preclinical study
Source: PLoS One. 2020 Apr 21;15(4):e0231488. doi: 10.1371/journal.pone.0231488 (PMC7173852; doi:10.1371/journal.pone.0231488)

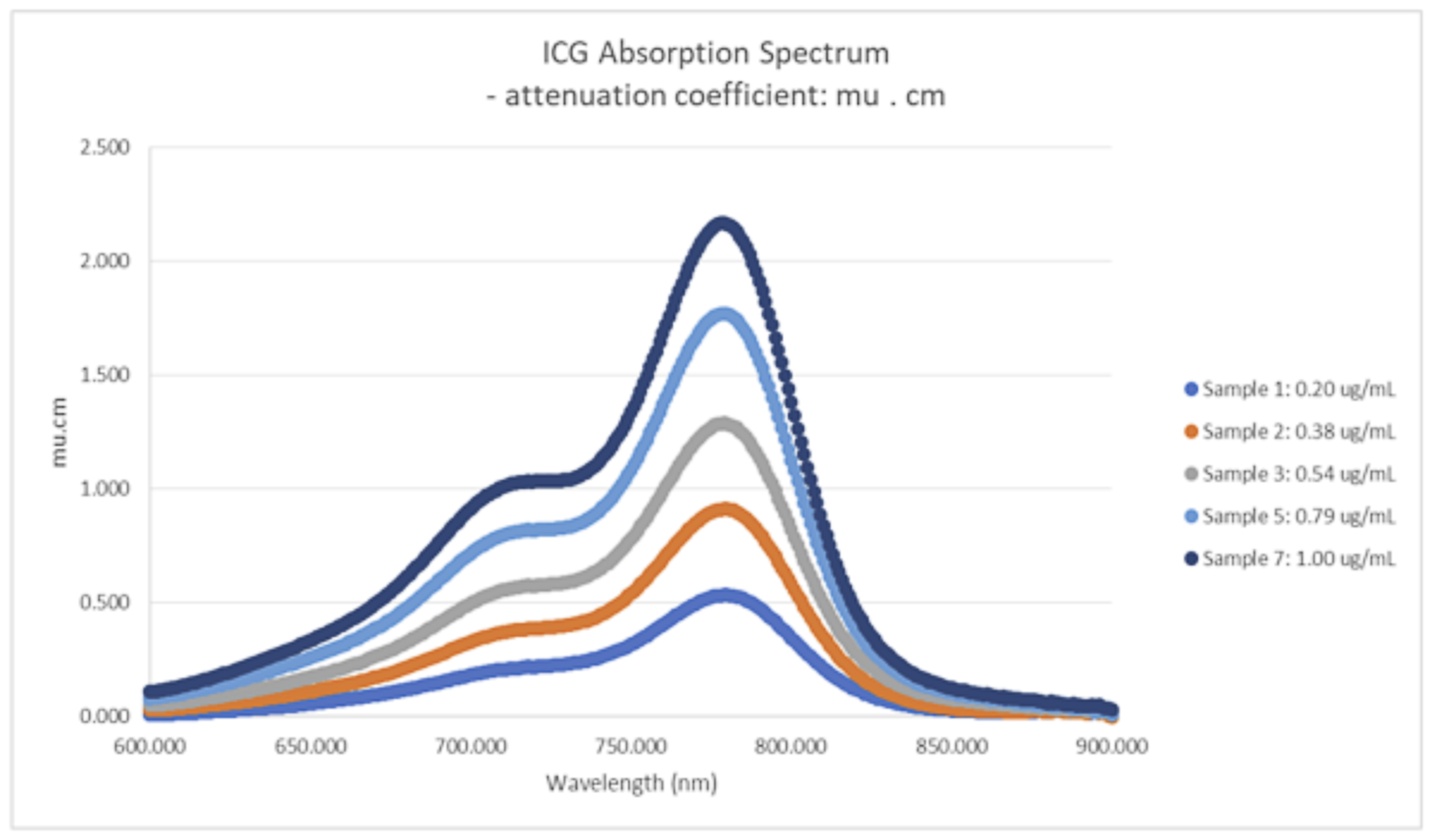

Supplement: S1 Fig — (TIF) [file pone.0231488.s001.tif]

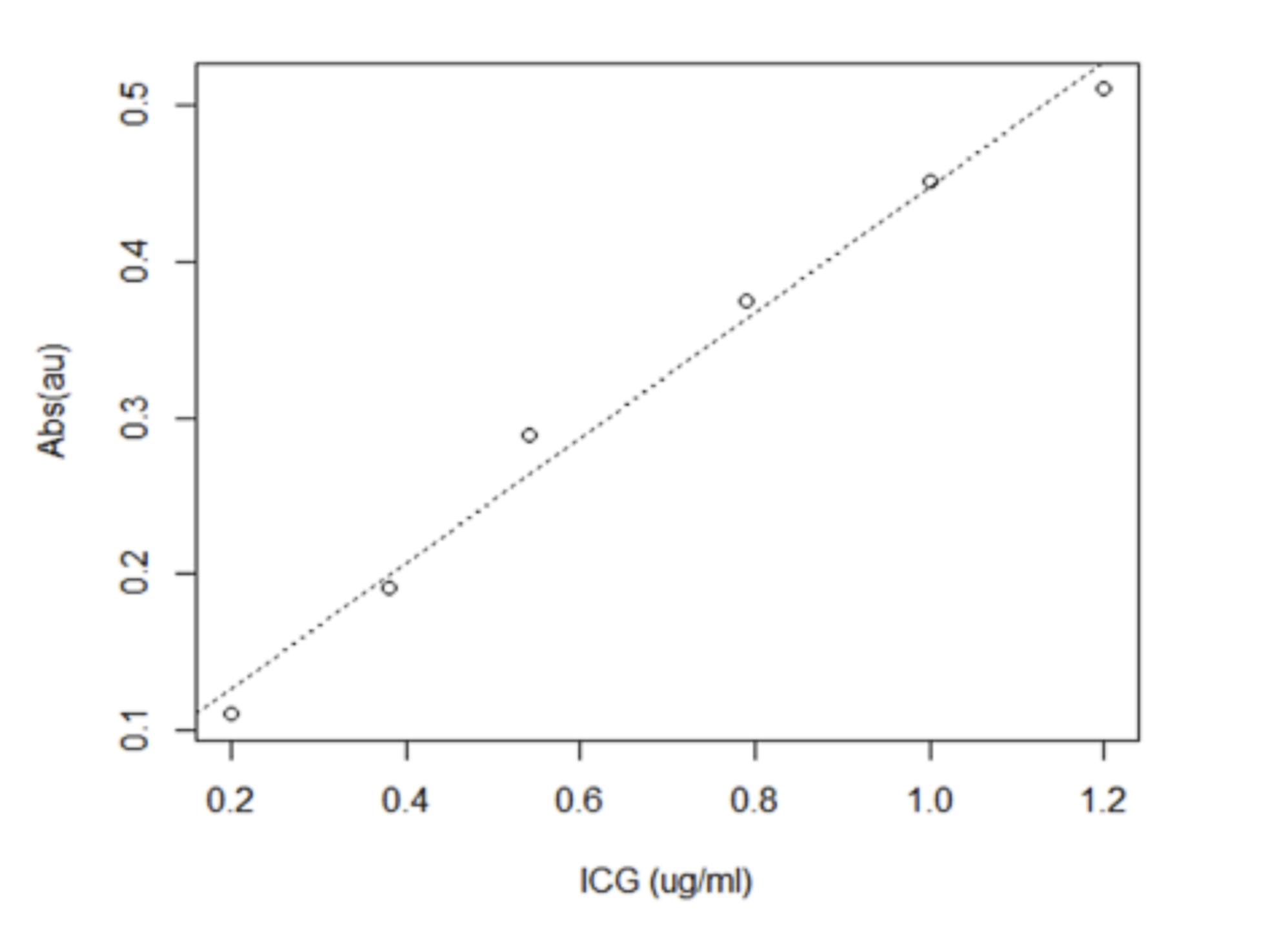

Supplement: S2 Fig — (TIF) [file pone.0231488.s002.tif]

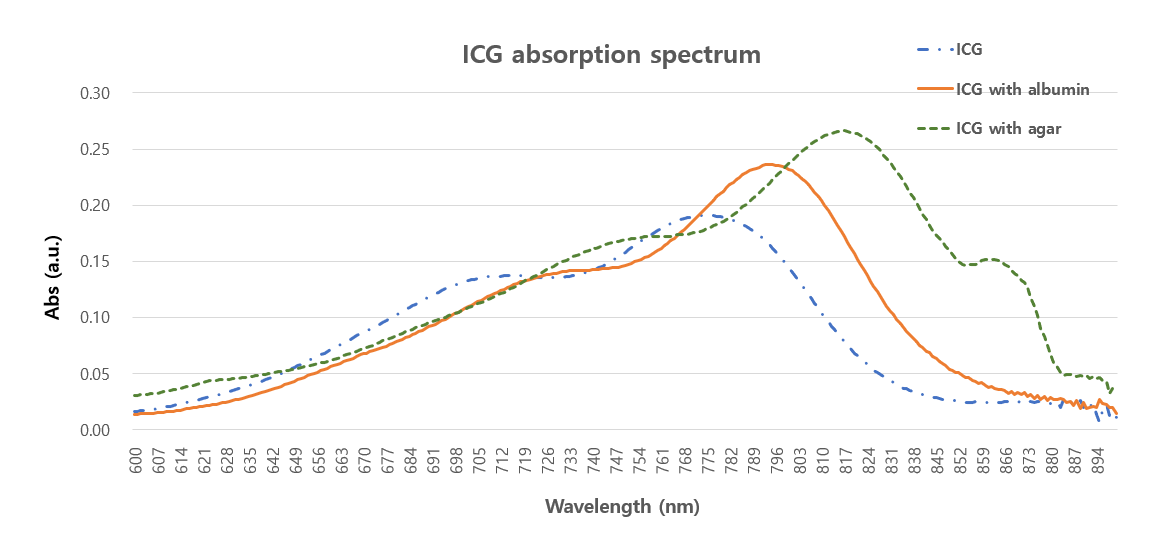

Supplement: S3 Fig — ICG absorbance in distilled water (blue, dashed line), bovine serum albumin (orange, solid line) and agar (green, dotted line). (TIF) [file pone.0231488.s003.tif]

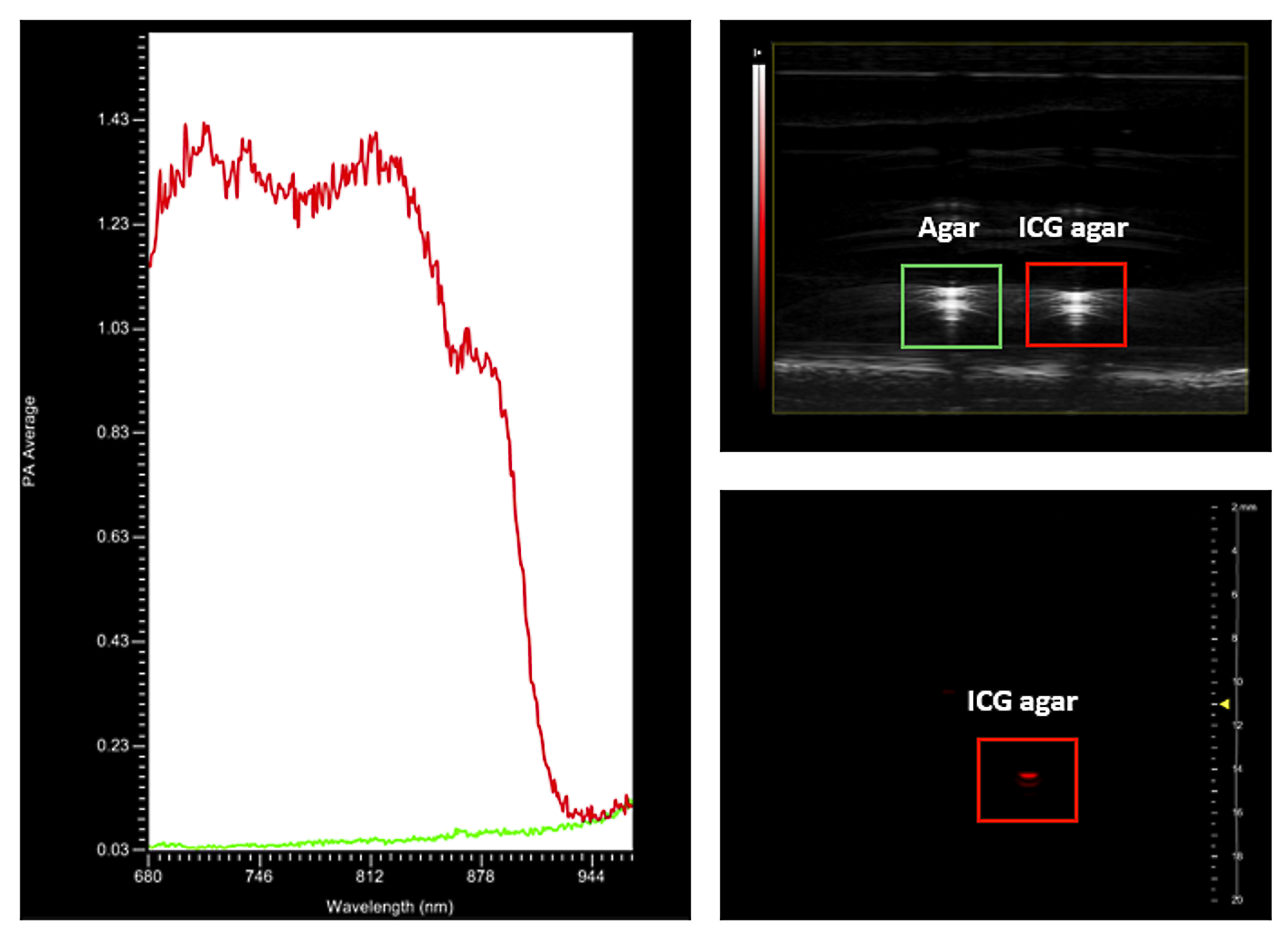

Supplement: S4 Fig — The PA spectrogram (left), US image (right upper) and PA image (right lower). Red line shows PA spectral change of ICG agar and green line shows that of agar itself in the right image. (TIF) [file pone.0231488.s004.tif]
