# Supplementary material for: Photoacoustic imaging to localize indeterminate pulmonary nodules: A preclinical study
Source: PLoS One. 2020 Apr 21;15(4):e0231488. doi: 10.1371/journal.pone.0231488 (PMC7173852; doi:10.1371/journal.pone.0231488)
Supplement: Attachment — Submitted filename: Respond to reviewer II.docx [file pone.0231488.s007.docx]

Reviewer #1: The author attempted to address, however, not satisfactorily, some of the questions raised earlier.

a) The quality of PA-images and spectra is not suitable for publication. If the visible quality of those images itself is difficult to perceive, how one can gain added value by using PA-image guided surgery in such ICG-agarose.

- Thanks for your comment. The PA image and spectra in current study were semi-automatically obtained from Vevo®LAZR system (Visualsonics, Fujifilm) and were submitted in this manuscript without any modification or manipulation to maintain the truth and reality of data. Unfortunately, authors was not able to get the PA images or spectra with more quality than those already submitted.

b) Manuscript lacks to bring out the advantage of using PA-image guided surgery.

- I partly agree with your comment. As far as I know, this study is a first attempt to explore the possibility of PA imaging technology combined with ICG-agar to detect or localize small lung nodule. Because of size limitation, I was not able to demonstrate clinically relevant lung cancer model using mice to prove the advantage of PA image guided surgery in this study. Recently, authors are developing lung cancer model using rabbit for further study. We hope that these results will be reporting soon.
